# Gaps in the implementation of antenatal syphilis detection and treatment in health facilities across sub-Saharan Africa

**DOI:** 10.1371/journal.pone.0198622

**Published:** 2018-06-01

**Authors:** Mufaro Kanyangarara, Neff Walker, Ties Boerma

**Affiliations:** 1 Department of International Health, Johns Hopkins Bloomberg School of Public Health, Baltimore, Maryland, United States of America; 2 Department of Community Health Sciences, University of Manitoba, Winnipeg, Manitoba, Canada; Harvard School of Public Health, UNITED STATES

## Abstract

**Background:**

Syphilis in pregnancy is an under-recognized public health problem, especially in sub-Saharan Africa which accounts for over 60% of the global burden of syphilis. If left untreated, more than half of maternal syphilis cases will result in adverse pregnancy outcomes including stillbirth and fetal loss, neonatal death, prematurity or low birth weight, and neonatal infections. Achieving universal coverage of antenatal syphilis screening and treatment has been the focus of the global campaign for the elimination of mother-to-child transmission of syphilis. However, little is known about the availability of antenatal syphilis screening and treatment across sub-Saharan Africa. The objective of this study was to estimate the ‘likelihood of appropriate care’ for antenatal syphilis screening and treatment by analyzing health facility surveys and household surveys conducted from 2010 to 2015 in 12 sub-Saharan African countries.

**Methods:**

In this secondary data analysis, we linked indicators of health facility readiness to provide antenatal syphilis detection and treatment from Service Provision Assessments (SPAs) and Service Availability and Readiness Assessments (SARAs) to indicators of ANC use from the Demographic and Health Surveys (DHS) to compute estimates of the ‘likelihood of appropriate care’.

**Results:**

Based on data from 5,593 health facilities that reported offering antenatal care (ANC) services, the availability of syphilis detection and treatment in ANC facilities ranged from 2% to 83%. The availability of syphilis detection and treatment was substantially lower in ANC facilities in West Africa compared to the other sub-regions. Levels of ANC attendance were high (median 94.9%), but only 27% of ANC attendees initiated care at less than 4 months gestation. We estimated that about one in twelve pregnant women received ANC early (<4 months) at a facility ready to provide syphilis detection and treatment (median 8%, range 7–32%). The largest implementation bottleneck identified was low health facility readiness, followed by timeliness of the first ANC visit.

**Conclusions:**

While access was fairly high, the low levels of likelihood of antenatal syphilis detection and treatment identified reinforce the need to improve the availability of syphilis rapid diagnostic tests and treatment and the timeliness of antenatal care-seeking across sub-Saharan Africa.

## Introduction

Globally, an estimated 5.6 million new syphilis cases occur every year, including one million cases among pregnant women [1,2]. Sub-Saharan Africa accounts for 63% of the global burden of syphilis in pregnancy, and the prevalence of antenatal syphilis seroreactivity ranges from 0% to 7.1% with a regional average estimated at 1.7% [2]. More than half of untreated maternal syphilis cases result in adverse pregnancy outcomes including stillbirth and fetal loss, neonatal death, prematurity or low birth weight, and neonatal infections [2,3]. Studies have also associated syphilis in pregnancy with an increased risk of acquiring and transmitting HIV and perinatal HIV transmission [4–6]. Early detection and treatment of syphilis in pregnancy is well-recognized as an effective strategy to reduce syphilis transmission and adverse pregnancy outcomes due to syphilis. In endemic countries, antenatal syphilis detection and treatment can reduce the number of stillbirths by 82%, preterm births by 64%, and neonatal deaths by 80% [7].

The World Health Organization (WHO) recommends syphilis screening at the first antenatal care (ANC) visit, ideally in the first trimester [8]. Antenatal syphilis screening has become simple, fast and inexpensive, even in settings with limited laboratory capacity, as a result of the development of point of care rapid diagnostics [8,9]. Timely treatment of seroreactive pregnant women with an intramuscular injection of penicillin G can prevent transmission of disease and syphilis-associated adverse pregnancy outcomes [7,10]. A recent meta-analysis found that syphilis screening and treatment during the first and second trimester of pregnancy compared to the third trimester reduced the risk of congenital syphilis by two thirds [11]. Despite the availability of simple diagnostic tools and highly effective and inexpensive treatment, screening and treatment of syphilis in pregnancy is not yet universal, and mother-to-child transmission (MTCT) of syphilis remains an under-recognized public health problem.

To address this problem, the WHO has called for the dual elimination of MTCT of syphilis and HIV [12–14]. Strategies are focused on ensuring sustained political commitment, improving access to and quality of maternal and newborn services, and universal screening and treatment of pregnant women and their partners [15–17]. Achieving elimination goals requires monitoring progress towards global targets on a range of indicators of health systems performance, from input indicators reflecting the availability and readiness of health facilities to provide essential screening and treatment, to outcome indicators reflecting rates of associated morbidity and mortality. However, routinely assessing progress towards elimination of MTCT of syphilis at national, regional and global levels has proved challenging, in part due to weak or non-existent routine health surveillance data reporting systems in many high burden countries. Despite the widespread adoption of policies for universal syphilis screening and treatment during pregnancy in many low- and middle-income countries, there is scant information on the availability, access to and coverage of antenatal syphilis detection and treatment. Several key indicators have been integrated within the Global AIDS Monitoring (GAM) system, and data from a number of countries are available through the WHO Global Health Observatory [18,19]. However, with limited data, tracking progress in the implementation of antenatal screening and treatment has primarily relied on modelling, and focused on estimation of the burden of disease [2].

Health facility surveys, complemented by household surveys offer an alternative approach to gain valuable insight on the availability, quality and uptake of reproductive, maternal, newborn and child health (RMNCH) interventions including antenatal syphilis detection and treatment [20–22]. Health facility surveys assess supply-side factors such as the availability of health services, essential medicines and commodities and human resources for health. Household surveys provide information on demand-side factors contributing to the universal coverage of essential health services. Linking household surveys to health facility surveys has been used to estimate population-level coverage of health interventions, particularly facility-based interventions not amenable to tracking by household surveys alone [23,24]. The framework of the linking approach addresses the need to consider both supply-side and demand-side factors driving coverage of health services. In this paper, we link household surveys and health facility surveys to assess the readiness of ANC facilities to provide syphilis detection and treatment to pregnant women and estimate the likelihood of appropriate care for syphilis detection and treatment across 12 sub-Saharan African countries. Based on our findings, we identify barriers to the implementation of antenatal screening and treatment of pregnant women and highlight opportunities to improve strategies for the elimination of MTCT of syphilis in sub-Saharan Africa.

## Methods

We conducted a secondary analysis of supply-side data obtained from two types of nationally representative cross-sectional health facility surveys, the Service Provision Assessment (SPA) and the Service Availability and Readiness Assessment (SARA). Both surveys use standardized data collection instruments to provide measures of availability and readiness of health facilities in a given country to provide essential services across several program areas including ANC. The availability of staff, guidelines, equipment, diagnostics, medicines and commodities is based on self-report and direct observation and verification. Further details on the sampling methods and survey procedures are available from final country survey reports [25,26]. This analysis focused on 12 countries in sub-Saharan Africa with a recent health facility survey, conducted between 2010 and 2015, and a household survey within +/- 2 years. Where multiple health facility surveys were available for the same country, the most recent survey was used. The 12 countries that met the inclusion criteria represented 4 sub-regions: Central Africa (Democratic Republic of Congo), East Africa (Kenya, Malawi, Tanzania, and Uganda), Southern Africa (Zimbabwe) and West Africa (Benin, Burkina Faso, Mauritania, Senegal, Sierra Leone and Togo). In 2012, the estimated number of pregnancies that occurred in these countries ranged from 122,246 in Mauritania to 3,036,898 in the Democratic Republic of Congo (Table 1). The total number of pregnancies with probable active syphilis infection was almost 200,000, representing 36% of the estimated burden in sub-Saharan Africa in 2012 [27].

**Table 1:** Characteristics of 12 countries included in the study sample.

Country	Annual number of pregnancies^a^	Number of pregnancies with probable active syphilis infections^a^	Neonatal mortality rate (per 1,000 livebirths)^b^	HIV prevalence (%)^c^	Antiretroviral therapy coverage for PMTCT (%)^d^
Benin	369,619	348	33	1.1	29
Burkina Faso	766,917	6,989	29	1.0	56
DRC	3,036,898	66,617	31	1.0	13
Kenya	1,622,428	11,391	25	5.9	55
Malawi	724,327	15,628	27	10.3	52
Mauritania	122,246	1,820	37	0.6	4
Senegal	492,601	8,377	24	0.6	31
Sierra Leone	233,917	2,260	38	1.7	76
Togo	201,475	1,659	24	5.2	95
Uganda	1,614,295	45,648	28	2.6	53
Tanzania	2,012,063	34,064	24	7.2	73
Zimbabwe	388,021	3,861	27	15.1	89

DRC: Democratic Republic of Congo. PMTCT: Prevention of mother to child HIV transmission.

^a^ Data retrieved from supplementary tables provided by Newman et al [27].

^b^ Estimates developed by the UN Inter-agency Group for Child Mortality Estimation [28].

^c^ Refers to the percentage of people ages 15–49 who are infected with HIV. Source: UNAIDS estimates [28].

^d^ Refers to the percentage of pregnant women with HIV who received antiretroviral therapy for PMTCT. Source: UNAIDS estimates [28].

To track progress in the implementation of antenatal syphilis detection and treatment we used a 3-step process. First, based on data from the health facility surveys, availability of syphilis detection for a given country was calculated as the percentage of health facilities providing ANC with observed availability of a rapid diagnostic test (RDT) on the day of assessment. Given WHO’s push for point of care diagnostics, in this study, facilities referring ANC clients or send blood samples elsewhere for screening were considered as not having syphilis screening available. The availability of syphilis detection and treatment was calculated as the percentage of health facilities providing ANC services with both a screening test for syphilis and treatment (benzathine penicillin or procaine penicillin, needles and syringes in stock).

Second, we analyzed demand-side data from publicly available nationally representative household surveys conducted as part of the Demographic and Health Surveys (DHS) program [29]. Information on patterns of antenatal care seeking for the most recent pregnancy, including where ANC was sought, when the first ANC visit occurred, and the ANC components received, is typically collected from women 15–49 years who gave birth in the 5 years preceding the survey. For this analysis, the household survey reference period was restricted to the three years preceding the survey. To reduce temporal misalignment, the estimation of the likelihood of appropriate care was only conducted for countries with a DHS conducted within (+/-) two years of the index health facility survey. No DHS was available within the required time frame for Burkina Faso and Mauritania. For the remaining 10 countries with a corresponding DHS within two years of the index health facility survey, we estimated the percentage of women who had a live birth in the three years preceding the survey who had at least one ANC visit with a skilled provider (ANC1+) and who had the first ANC visit at less than 4 months gestation.

Third, estimates of the likelihood of appropriate care were calculated by multiplying indicators of service utilization (ANC1+ and timing of first ANC visit) by indicators of health facility readiness at the stratum level. As service utilization and health facility readiness vary within countries, linking was conducted at the stratum level which was defined by health facility type (e.g. health post, health center and hospital) and managing authority (public/non-public). As women who attended multiple ANC visits can report multiple sources of ANC in the DHS, we made the simplifying assumption that these women sought care at the highest level of facility type reported. The estimates of likelihood of appropriate care for each country were disaggregated by timing of the first ANC visit (categorized as <4, 4–6, ≥7 months). Women who attended at least one ANC visit at a health facility with syphilis detection and treatment available were classified as having a high likelihood of appropriate care, while those who sought ANC at a health facility with only syphilis detection available were classified as having a moderate likelihood of appropriate care. Women who sought ANC at a health facility that did not have the necessary diagnostics in stock were classified as having a low likelihood of appropriate care due to low health facility readiness. All other women did not seek any ANC and were considered classified as having no likelihood of appropriate care. This classification formed the basis for our identification of three bottlenecks in the implementation pathway of antenatal syphilis detection and treatment: access, timeliness, and health facility readiness.

All analyses were conducted at the country level and took into account the sampling design (survey-specific stratum, cluster and sampling weights). Because of important differences in epidemiological and programmatic context, we grouped country-specific results by sub-region. All analyses were conducted in STATA 14.2 (College Station, Texas).

## Results

### Availability of syphilis detection and treatment

**Fig 1:**
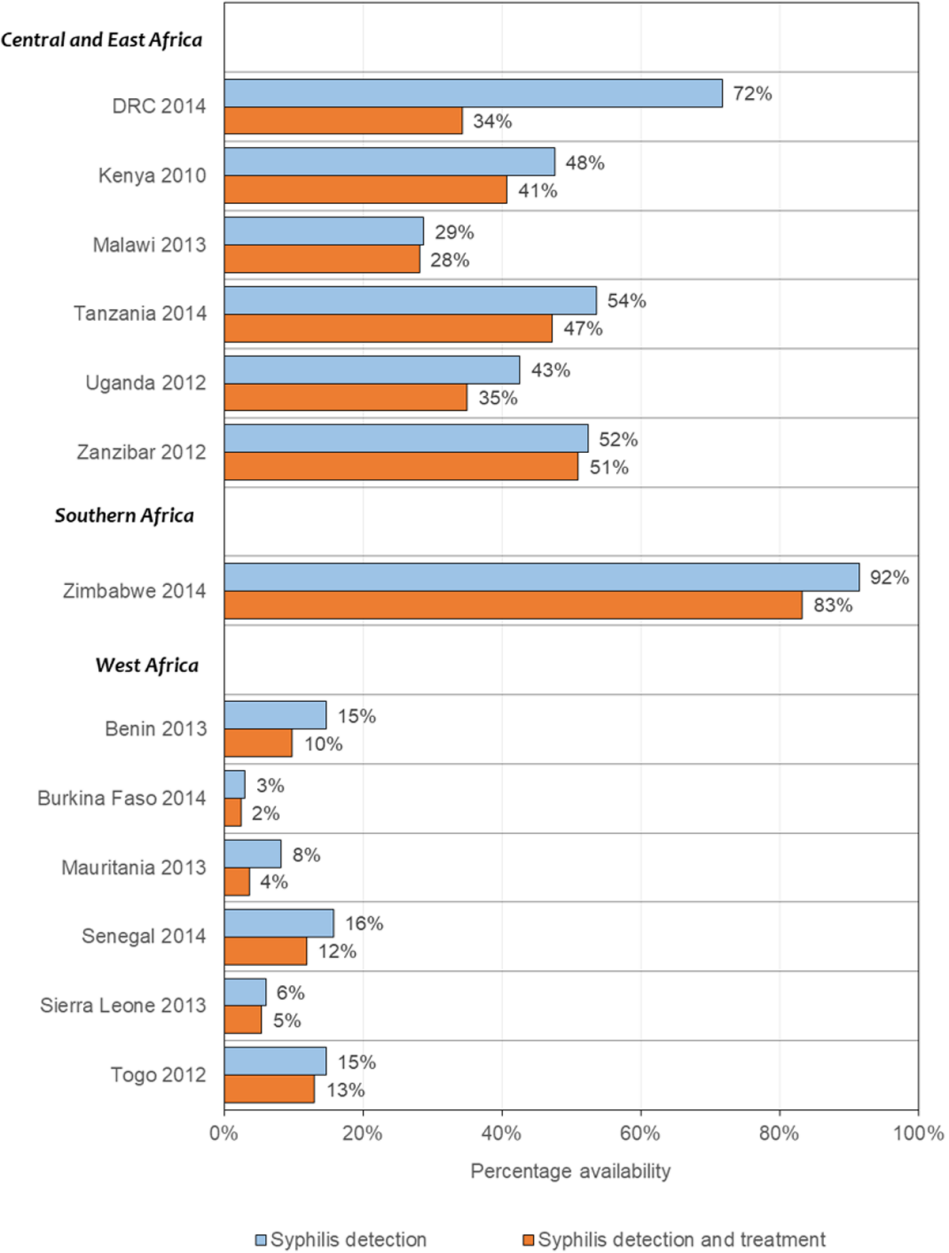
Availability of syphilis detection and treatment in health facilities offering antenatal care (ANC) in 12 sub-Saharan African countries, 2010–2015. DRC: Democratic Republic of Congo.

A total of 6,991 health facilities were sampled in the 12 sub-Saharan African countries during 2010 to 2015; health facility survey sample sizes ranged from 95 (Uganda) to 1,555 (Democratic Republic of Congo, Table 2). A subset of 5,593 health facilities that reported offering ANC were included in this analysis. Diagnostic capacity for syphilis at health facilities offering ANC varied across countries, ranging from 3% in Burkina Faso to 92% in Zimbabwe (Fig 1). In general, diagnostic capacity was lower in health facilities in West Africa relative to the other sub-regions (range 3% - 15%). By and large, most health facilities with diagnostic capacity also had syphilis treatment available (range 44% - 98%). However, in DRC, while 72% of facilities offering ANC had syphilis detection available, only half of those also had treatment available.

**Table 2:** Health facility and household surveys included.

	Health facility survey						Household survey							
**Country**	**Year**	**Type**	**Total number of health facilities**	**Facilities offering ANC services (%)**	ANC facilities with staff trained in ANC*(%)	**ANC facilities with guidelines on ANC (%)**	**Year**	**Type**	**Total number of households sampled**	**ANC1+** **(%)**	**ANC4+ (%)**	**Early ANC enrollment (%)**^**1**^	**Median months of pregnancy at 1**^**st**^ **ANC visit**	**Blood sample taken (%)**
Benin	2013	SARA	189	84	49.3	70.8	2011/12	DHS	17,422	86.0	58.3	48.3	3.8	81.3
Burkina Faso	2014	SARA	766	88	73.4	92.1	-	-		-	-	-	-	-
DRC	2014	SARA	1,555	74	45.3	47.3	2013/14	DHS	18,171	88.5	47.2	16.7	5.4	61.4
Kenya	2010	SPA	695	81	78.5	63.5	2008/9	DHS	9,057	91.5	45.7	13.5	5.8	82.7
Malawi	2013	SPA	977	66	39.1	71.1	2015/16	DHS	26,361	99.1	49.5	23.8	4.9	92.5
Mauritania	2013	SARA	232	72	45.3	54.4	-	-		-	-	-	-	-
Senegal	2014	SPA	452	77	46.8	62.0	2014	DHS	4,231	96.1	47.1	57.8	3.6	85.5
Sierra Leone	2013	SARA	455	94	88.9	85.0	2013	DHS	12,629	97.5	75.8	44.7	4.1	89.3
Tanzania	2014/15	SPA	1,200	86	30.9	60.7	2015/16	DHS	12,563	97.9	49.2	23.1	5.0	87.3
Togo	2012	SARA	100	92	31.4	77.3	2013/14	DHS	9,549	93.1	56.4	27.0	4.9	87.4
Uganda	2012	SARA	95	85	64.8	59.6	2011	DHS	9,033	94.9	47.2	20.7	5.2	83.4
Zimbabwe	2014	SARA	275	95	99.2	96.2	2015	DHS	10,534	92.0	74.1	37.4	4.5	98.7
Median			453.5	84.5	48.1	67.2				94.9	49.2	27	4.9	85.5

- No corresponding household survey was available for Mauritania and Burkina Faso.

^1^ Early ANC enrollment was defined as first ANC visit at less than four months gestation.

* Staff trained was defined as at least one staff member trained in any aspect of ANC in the previous 2–3 years.

### Timing and coverage of antenatal care

Across the 12 countries, nearly all pregnant women made at least one ANC visit (median 94.9%; Table 2). Benin, Democratic Republic of Congo, Kenya, Togo and Zimbabwe had ANC1+ coverage below 95%. The drop off in ANC attendance from coverage of at least one visit (ANC1+) to coverage of 4 or more visits (ANC4+) ranged from 18 percentage points in Zimbabwe to 62 percentage points in Burkina Faso. There were also substantial differences in the timing of the first ANC visit, with the median months of gestation at first ANC visit varying from 3.6 months in Senegal to 5.8 months in Kenya. While the recommendation is that all pregnant women are screened for syphilis during the first ANC visit in the first trimester, the percentage of women attending the first ANC visit at less than four months was low in all countries, with considerable variability (median 27%; range:13.5% - 57.8%; Table 2). For example, in Malawi, while 99% of all pregnant women attended at least one ANC visit, only 23.8% attended ANC early enough to experience the maximum benefit of treatment on the risk of adverse outcome due to syphilis.

### Likelihood of appropriate care for syphilis detection and treatment

**Fig 2:**
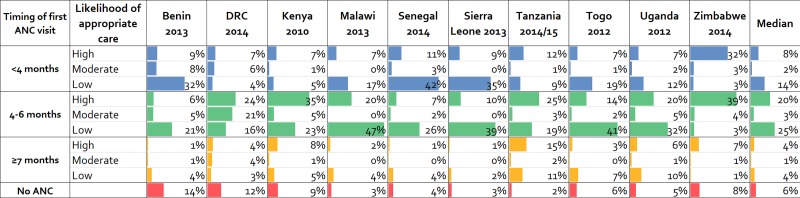
Likelihood of appropriate care for antenatal syphilis detection and treatment in 10 sub-Saharan African countries, 2010–2015. ANC: antenatal care. DRC: Democratic Republic of Congo. LAC: likelihood of appropriate care.

Based on the linking approach, we estimated that across countries one in twelve women received ANC at a facility ready to provide syphilis detection and treatment during the first three months of pregnancy (high ‘likelihood of appropriate care’; median 8%; range: 7% - 32%) (Fig 2). If only the availability of syphilis detection was considered (high and moderate ‘likelihood of appropriate care’ combined), then one in ten women received ANC during the first three months of pregnancy at a facility ready to provide syphilis screening (median 10%; range: 7% - 35%). Among women who received ANC at a facility ready to provide syphilis detection, one in eight initiated ANC after the first six months of pregnancy (median 12%; range: 3% - 28%). Due to delayed ANC seeking, these women may have missed the opportunity to receive the maximum benefit of early syphilis detection and treatment. Additionally, among women who initiated ANC in the first six months, more than half (median 54%; range 7% - 79%) missed the opportunity to be screened for syphilis as a result of low health facility readiness (i.e. no availability of syphilis RDTs).

## Discussion

Despite the widespread adoption of antenatal screening and treatment of syphilis as the main strategy for the prevention of MTCT of syphilis, we found suboptimal implementation strength across 12 sub-Saharan African countries. The global campaign to eliminate MTCT of syphilis has targeted achievement of at least 95% on three process indicators: coverage of ANC1+, coverage of syphilis testing of pregnant women and treatment of syphilis-seropositive pregnant women [16]. Using our approach linking supply-side data from health facility surveys and demand-side data from household surveys, our estimates of the percentage of pregnant women who received early ANC at a facility with a syphilis RDT and penicillin treatment available (high likelihood of appropriate care) fell well below 95% coverage target (range 7% - 32%). We identified three bottlenecks in the implementation of appropriate syphilis detection and treatment during pregnancy: access, timeliness, and health facility readiness.

ANC is a key platform for the delivery of evidence-based RMNCH interventions. Access to ANC has improved in recent years [30]. Notably, 6 out of the 10 sub-Saharan Africa countries with recent surveys in this study had attained ANC1+ coverage levels above the 95% target. However, recent evidence suggests low coverage of ANC interventions resulting in substantial missed opportunities to provide quality health services [21,31]. With respect to antenatal syphilis screening and treatment, the timing of the first ANC visit makes a significant difference on the risk of adverse pregnancy outcomes due to syphilis [11]. In this study the median gestational age at first ANC visit ranged from 3.6 months in Senegal to 5.8 months in Kenya. The timeliness of ANC was identified as a bottleneck in the implementation of appropriate syphilis detection and treatment. Between 25% and 85% of pregnant women did not seek ANC until 4 months or later. To fully benefit from the early detection and treatment, there is need for effective strategies to promote early ANC initiation.

Health facility readiness was another major bottleneck. Across countries, a median of 54% of pregnant women who sought ANC in the first six months could not access syphilis detection and treatment due to inadequate supply of syphilis tests and treatment in ANC facilities, representing substantial missed opportunities for the delivery of high impact interventions to pregnant women seeking ANC early. The low availability of syphilis detection and treatment to ANC attendees identified in this study supports the findings that improving access to ANC does not guarantee the delivery of quality RMNCH services [21,31,32]. The availability of syphilis tests and treatment at health facilities across sub-Saharan Africa was sub-optimal and varied by country. For instance, in Zimbabwe, syphilis tests and treatment were available in most facilities offering ANC (83%). By contrast, syphilis tests and treatment were in low supply in ANC facilities across West African countries (Benin 10%, Burkina Faso 2%, Mauritania 4%, Senegal 12%, Sierra Leone 5% and Togo 13%). This difference in health facility readiness may reflect relatively lower burden of syphilis, poorer health infrastructures and few health system resources in West Africa [33]. The difference may also be explained by increased attention due to higher burden of HIV and the integration of syphilis interventions into existing ANC and prevention of MTCT of HIV programs in Zimbabwe and other countries in Southern Africa [12,34]. There are too few Southern African countries to systematically assess this explanation. However, UNAIDS estimates of HIV prevalence in 2012 for the 6 West African countries ranged from 0.6% - 2.6% compared to 15.1% for Zimbabwe and 5.9% - 10.3% for the 4 East African countries [35].

Greater efforts are needed to strengthen implementation, improve the quality of RMNCH services and accelerate progress towards elimination of MTCT of syphilis. Continued efforts to routinely measure and track progress in universal screening and treatment of syphilis during pregnancy and more broadly, key RMNCH interventions are needed [23,24]. The inclusion of indicators for syphilis in pregnancy using a unified system such as the UNAIDS GAM system is an important step to support routine reporting and the collation of data from surveillance systems in low- and middle-income countries. While efforts should be made to establish and improve surveillance systems in the long term, the expansion in geographic scope and frequency of health facility surveys such as the SPA and SARA can provide information to fill crucial data gaps. Health facility surveys are useful for monitoring and addressing deficiencies in the availability and quality of service provision [36], and linked with household surveys can facilitate the estimation of coverage of interventions and identification of implementation bottlenecks [20,21,23,37].

There are several limitations to this analysis. First, the definition of high likelihood of appropriate care used was the percentage of women who attended ANC in the first three months at a facility with syphilis detection and treatment available. While the availability of basic amenities, equipment, diagnostics, medicines and commodities is a prerequisite for the delivery of antenatal syphilis screening and treatment, it does not guarantee receipt of care. Information on the receipt of syphilis screening and treatment is not typically available, therefore our estimates may overestimate ‘true coverage’. We did not account for factors such as provider knowledge or other health system factors that may hinder the actual receipt of interventions. Estimates of the likelihood of appropriate care are likely biased upwards. Second, the present analysis linked nationally representative household surveys and health facility surveys covering different survey reference periods. The availability of medicines and diagnostics can vary dramatically over a short time period due to the complexity of logistics, availability of penicillin, and seasonality. While the present study did not assess drug stock-outs, several countries in sub-Saharan Africa have experienced stock-outs of benzathine penicillin [38]. Therefore, while data from health facility surveys may be representative of health system at one point in time, such data may not represent the current state. More periodic health facility surveys can provide timely information necessary to guide national and sub-national policy and program prioritization; health facility surveys need to be conducted in more countries and at more regular interval. Most of the health facility surveys included in the present analysis represent East and West Africa. Findings from this analysis should be not be considered as representative of sub-Saharan Africa, but rather as evidence to guide the ongoing campaign to end MTCT of syphilis. Lastly, the effectiveness of syphilis treatment during pregnancy is not a dichotomous variable. While the effectiveness of treatment decreases with increasing duration of pregnancy, treatment later in pregnancy is known to have some effect on health outcomes. The greatest effects on the prevention of congenital syphilis are observed when given before approximately 21–24 weeks [7,39], when the fetal immune system is still immature and has not yet developed an (adverse) response to the syphilis infection [40]. Evidence of the population-level effectiveness of syphilis screening and treatment by duration of pregnancy is currently limited to a meta-analysis which compared the effects during the first and second trimester of pregnancy compared to the third trimester [11]. In our study, we defined timeliness as the first visit occurring before 4 months since treatment of syphilis as early as possible in pregnancy is highly desirable. As women who attend ANC after the first three months of pregnancy could still benefit from screening and treatment, findings were disaggregated by timing of first ANC visit.

This study suggests low levels of availability of antenatal syphilis screening and treatment across 12 sub-Saharan African countries, albeit with wide variability in progress towards the elimination of MTCT of syphilis. Deficiencies in access, health facility readiness and timeliness of ANC identified represent opportunities to improve the coverage and quality of syphilis detection and treatment to pregnant women. Progress towards the elimination of MTCT of syphilis depends on sustained high levels of ANC uptake, improved timeliness of care-seeking, and increased availability of syphilis detection and treatment at health facilities across sub-Saharan Africa.

## References

[1] Newman L, Rowley J, Vander Hoorn S, Wijesooriya NS, Unemo M, Low N, et al Global Estimates of the Prevalence and Incidence of Four Curable Sexually Transmitted Infections in 2012 Based on Systematic Review and Global Reporting. PLoS One. 2015;10(12):1–17. 10.1371/journal.pone.0143304 PMC4672879 26646541

[2] Wijesooriya NS, Rochat RW, Kamb ML, Turlapati P, Temmerman M, Broutet N, et al Global burden of maternal and congenital syphilis in 2008 and 2012: a health systems modelling study. Lancet Glob Heal. 2016;4(8):e525–33. 10.1016/S2214-109X(16)30135-8 PMC6759483 27443780

[3] Gomez GB, Kamb ML, Newman LM, Mark J, Broutet N, Hawkes SJ. Untreated maternal syphilis and adverse outcomes of pregnancy: a systematic review and meta-analysis. Bull World Health Organ. 2013;91:217–26. doi: 10.2471/BLT.12.107623 23476094 10.2471/BLT.12.107623 PMC3590617

[4] Ward H, Ronn M. Contribution of sexually transmitted infections to the sexual transmission of HIV. Curr Opin HIV AIDS. 2010 7;5(4):305–10. doi: 10.1097/COH.0b013e32833a8844 20543605 10.1097/COH.0b013e32833a8844 PMC2923028

[5] Mwapasa V, Rogerson SJ, Kwiek JJ, Wilson PE, Milner D, Molyneux ME, et al Maternal syphilis infection is associated with increased risk of mother-to-child transmission of HIV in Malawi. AIDS. 2006;20(14). 10.1097/01.aids.0000244206.41500.27 16954728

[6] Sexton J, Garnett G, Røttingen J-A. Metaanalysis and Metaregression in Interpreting Study Variability in the Impact of Sexually Transmitted Diseases on Susceptibility to HIV Infection. Sex Transm Dis. 2005;32(6). 10.1097/01.olq.0000154504.54686.d1 15912081

[7] Blencowe H, Cousens S, Kamb M, Berman S, Lawn JE. Lives Saved Tool supplement detection and treatment of syphilis in pregnancy to reduce syphilis related stillbirths and neonatal mortality. BMC Public Health. 2011 4;11(3):S9. 10.1186/1471-2458-11-S3-S9 PMC3231915 21501460

[8] World Health Organization. WHO guideline on syphilis screening and treatment for pregnant women. Geneva: World Health Organization; 2017. 29757595

[9] Mabey DC, Sollis KA, Kelly HA, Benzaken AS, Bitarakwate E, Changalucha J, et al Point-of-Care Tests to Strengthen Health Systems and Save Newborn Lives: The Case of Syphilis. PLOS Med. 2012;9(6):1–6. 10.1371/journal.pmed.1001233 PMC3373627 22719229

[10] Watson-Jones D. Syphilis in pregnancy in Tanzania. II. The effectiveness of antenatal syphilis screening and single-dose benzathine penicillin treatment for the prevention of adverse pregnancy outcomes. J Infect Dis. 2002;186. 10.1086/342951 12232835

[11] Hawkes SJ, Gomez GB, Broutet N. Early Antenatal Care: Does It Make a Difference to Outcomes of Pregnancy Associated with Syphilis? A Systematic Review and Meta-Analysis. PLoS One. 2013;8(2):1–7. 10.1371/journal.pone.0056713 PMC3585307 23468875

[12] Newman Owiredu M, Newman L, Nzomo T, Conombo Kafando G, Sanni S, Shaffer N, et al Elimination of mother-to-child transmission of HIV and syphilis: A dual approach in the African Region to improve quality of antenatal care and integrated disease control. Int J Gynaecol Obstet. 2015;130:S27–31. doi: 10.1016/j.ijgo.2015.04.010 25963908 10.1016/j.ijgo.2015.04.010

[13] Pan American Health Organization, UNICEF. Regional initiative for the elimination of mother-to-child transmission of HIV and congenital syphilis in Latin America and the Caribbean: regional monitoring strategy. Washington, DC; 2010.

[14] World Health Organization. Global guidance on criteria and processes for validation: elimination of mother-to-child transmission of HIV and syphilis, 2nd edition. Geneva; 2017.

[15] World Health Organization. Global health sector strategy on Sexually Transmitted Infections, 2016–2021. Geneva; 2016.

[16] World Health Organization Elimination of mother-to-child transmission (EMTCT) of HIV and syphilis: Global guidance on criteria and processes for validation. Geneva: WHO. Geneva; 2014.

[17] WHO | World Health Organization. Prevention of Mother-to-Child-Transmission of Syphilis (Congenital Syphilis) [Internet]. Available from: http://www.who.int/reproductivehealth/congenital-syphilis/en/

[18] World Health Organization. Global Health Observatory:(GHO). World Health Organization; 2014.

[19] UNAIDS. Global AIDS Monitoring 2017. 2016.

[20] Do M, Micah A, Brondi L, Campbell H, Marchant T, Eisele T, et al Linking household and facility data for better coverage measures in reproductive, maternal, newborn, and child health care: systematic review. J Glob Health. 2016 12 3;6(2):20501. 10.7189/jogh.06.020501 PMC5012234 27606060

[21] Kanyangarara M, Walker N, Munos M. Quality of Antenatal Care Service Provision in Health Facilities across Sub-Saharan Africa: Evidence from Nationally Representative Health Facility Assessments. J Glob Health. 10.7189/jogh.07.021101 PMC5680531 29163936

[22] Baker U, Okuga M, Waiswa P, Manzi F, Peterson S, Hanson C. Bottlenecks in the implementation of essential screening tests in antenatal care: Syphilis, HIV, and anemia testing in rural Tanzania and Uganda. Int J Gynaecol Obstet. 2015;130 Suppl:S43–50. 26054252 10.1016/j.ijgo.2015.04.017

[23] Munos MK, Stanton CK, Bryce J. Improving coverage measurement for reproductive, maternal, neonatal and child health: gaps and opportunities. J Glob Health. 2017 6;7(1):10801. 10.7189/jogh.07.010801 PMC5460400 28607675

[24] Bryce J, Arnold F, Blanc A, Hancioglu A, Newby H, Requejo J, et al Measuring Coverage in MNCH: New Findings, New Strategies, and Recommendations for Action. PLoS Med. 2013;10(5):1–9. 10.1371/journal.pmed.1001423 PMC3646206 23667340

[25] WHO | Service availability and readiness assessment (SARA) [Internet]. [cited 2017 Oct 31]. Available from: http://www.who.int/healthinfo/systems/sara_introduction/en/

[26] The DHS Program—Service Provision Assessments (SPA) [Internet]. [cited 2016 Oct 31]. Available from: http://dhsprogram.com/What-We-Do/Survey-Types/SPA.cfm

[27] Newman L, Kamb M, Hawkes S, Gomez G, Say L, Seuc A, et al Global Estimates of Syphilis in Pregnancy and Associated Adverse Outcomes: Analysis of Multinational Antenatal Surveillance Data. PLOS Med. 2013;10(2):1–10. 10.1371/journal.pmed.1001396 PMC3582608 23468598

[28] World Bank. World Bank Open Data [Internet]. Available from: https://data.worldbank.org/

[29] The DHS Program—Available Datasets [Internet]. [cited 2016 Dec 8]. Available from: http://dhsprogram.com/data/available-datasets.cfm

[30] Victora CG, Requejo JH, Barros AJD, Berman P, Bhutta Z, Boerma T, et al Countdown to 2015: a decade of tracking progress for maternal, newborn, and child survival. Lancet. 2016;387(10032):2049–59. doi: 10.1016/S0140-6736(15)00519-X 26477328 10.1016/S0140-6736(15)00519-X PMC7613171

[31] Hodgins S, D’Agostino A. The quality-coverage gap in antenatal care: toward better measurement of effective coverage. Glob Heal Sci Pract. 2014;2(2):173–81. 10.9745/GHSP-D-13-00176 PMC4168625 25276575

[32] Kruk ME, Leslie HH, Verguet S, Mbaruku GM, Adanu RMK, Langer A. Quality of basic maternal care functions in health facilities of five African countries: an analysis of national health system surveys. Lancet Glob Heal. 2016;(16):1–11. 10.1016/S2214-109X(16)30180-2 27670090

[33] Agyepong IA, Kwamie A, Frimpong E, Defor S, Ibrahim A, Aryeetey GC, et al Spanning maternal, newborn and child health (MNCH) and health systems research boundaries: conducive and limiting health systems factors to improving MNCH outcomes in West Africa. Heal Res Policy Syst. 2017 7;15(1):54. 10.1186/s12961-017-0212-x PMC5516848 28722556

[34] Strasser S, Bitarakwate E, Gill M, Hoffman HJ, Musana O, Phiri A, et al Introduction of rapid syphilis testing within prevention of mother-to-child transmission of HIV programs in Uganda and Zambia: a field acceptability and feasibility study. J Acquir Immune Defic Syndr. 2012 11;61(3):e40–6. doi: 10.1097/QAI.0b013e318267bc94 22820810 10.1097/QAI.0b013e318267bc94

[35] UNAIDS. AIDSinfo [Internet]. Available from: http://aidsinfo.unaids.org/.

[36] Neill KO, Takane M, Sheffel A, Abou-zahr C, Boerma T. Monitoring service delivery for universal health coverage: the Service Availability and Readiness Assessment. Bull World Health Organ. 2013;(9):923–31. doi: 10.2471/BLT.12.116798 24347731 10.2471/BLT.12.116798 PMC3845262

[37] Baker U, Peterson S, Marchant T, Mbaruku G, Temu S, Manzi F, et al Identifying implementation bottlenecks for maternal and newborn health interventions in rural districts of the United Republic of Tanzania. Bull World Health Organ. 2015;93(6):380–9. doi: 10.2471/BLT.14.141879 26240459 10.2471/BLT.14.141879 PMC4450702

[38] Nurse-Findlay S, Taylor MM, Savage M, Mello MB, Saliyou S, Lavayen M, et al Shortages of benzathine penicillin for prevention of mother-to-child transmission of syphilis: An evaluation from multi-country surveys and stakeholder interviews. PLOS Med. 2017;14(12):1–18. 10.1371/journal.pmed.1002473 PMC5744908 29281619

[39] Gust DA, Levine WC, St. Louis ME, Braxton J, Berman SM. Mortality Associated With Congenital Syphilis in the United States, 1992–1998. Pediatrics. 2002;109(5):e79—e79. 11986485 10.1542/peds.109.5.e79

[40] Berman SM. Maternal syphilis: pathophysiology and treatment. Bull World Health Organ. 2004;82(6):433–8. 15356936 PMC2622860

